# Whole-genome bisulfite sequencing in systemic sclerosis provides novel targets to understand disease pathogenesis

**DOI:** 10.1186/s12920-019-0602-8

**Published:** 2019-10-24

**Authors:** Tianyuan Lu, Kathleen Oros Klein, Inés Colmegna, Maximilien Lora, Celia M. T. Greenwood, Marie Hudson

**Affiliations:** 1 Lady Davis Institute for Medical Research, Jewish General Hospital, 3755 Côte Sainte-Catherine Road, Montreal, H3T 1E2 Canada; 2 Quantitative Life Sciences Program, McGill University, Montreal, Canada; 3 Division of Rheumatology, Department of Medicine, McGill University, Montreal, Canada; 4 Department of Epidemiology, Biostatistics & Occupational Health, McGill University, Montreal, Canada; 5 Gerald Bronfman Department of Oncology, McGill University, Montreal, Canada; 6 Department of Human Genetics, McGill University, Montreal, Canada

**Keywords:** Systemic sclerosis, Whole-genome bisulfite sequencing, Differential methylation, Pathway analysis, SNP-CpG association

## Abstract

**Background:**

Systemic sclerosis (SSc) is a rare autoimmune connective tissue disease whose pathogenesis remains incompletely understood. Increasing evidence suggests that both genetic susceptibilities and changes in DNA methylation influence pivotal biological pathways and thereby contribute to the disease. The role of DNA methylation in SSc has not been fully elucidated, because existing investigations of DNA methylation predominantly focused on nucleotide CpGs within restricted genic regions, and were performed on samples containing mixed cell types.

**Methods:**

We performed whole-genome bisulfite sequencing on purified CD4+ T lymphocytes from nine SSc patients and nine controls in a pilot study, and then profiled genome-wide cytosine methylation as well as genetic variations. We adopted robust statistical methods to identify differentially methylated genomic regions (DMRs). We then examined pathway enrichment associated with genes located in these DMRs. We also tested whether changes in CpG methylation were associated with adjacent genetic variation.

**Results:**

We profiled DNA methylation at more than three million CpG dinucleotides genome-wide. We identified 599 DMRs associated with 340 genes, among which 54 genes exhibited further associations with adjacent genetic variation. We also found these genes were associated with pathways and functions that are known to be abnormal in SSc, including Wnt/β-catenin signaling pathway, skin lesion formation and progression, and angiogenesis.

**Conclusion:**

The CD4+ T cell DNA cytosine methylation landscape in SSc involves crucial genes in disease pathogenesis. Some of the methylation patterns are also associated with genetic variation. These findings provide essential foundations for future studies of epigenetic regulation and genome-epigenome interaction in SSc.

## Background

Systemic sclerosis (SSc) is a chronic autoimmune disease characterized by skin and visceral organ fibrosis [1, 2]. It is associated with high morbidity and mortality [3–5]. Aside from autologous hematopoietic stem cell transplant, which is associated with serious treatment related toxicities and is indicated for a small proportion of patients, no therapies have been shown to meaningfully modify disease progression [6]. The pathogenesis of SSc remains to be fully elucidated and this is key to identifying novel therapeutic targets.

Increasing evidence suggests that genetic risk factors are strongly associated with SSc. Though the human leukocyte antigen (HLA) class II region is traditionally considered the most genetically associated with SSc [7–9], non-HLA loci and corresponding genes have also been identified [10, 11]. However, these SSc-susceptibility loci are estimated to account for only a small proportion of the disease risk [11].

In recent years, epigenetic dysregulation, including DNA methylation, histone modifications and microRNA expression, have been associated with SSc pathogenesis [12]. In particular, several studies have demonstrated that aberrant DNA methylation patterns at CpG dinucleotides are associated with altered expression levels of key genes related to SSc [12–14]. These findings complement the known genetic associations and provide tantalizing additional clues to the etiology of SSc.

However, the majority of epigenetic studies in SSc to date have three important shortcomings. First, most studies of DNA methylation used assays that measure DNA methylation in restricted genic regions. Since these regions constitute a small proportion of the genome, changes in DNA methylation patterns are yet to be discovered in the remaining regions, both genic and intergenic. Second, though studies of other diseases have unraveled the non-negligible role of CHG/CHH methylation (where “H” implies any nucleotide other than G), particularly in cancer [15, 16], no studies have investigated CHG/CHH methylation in SSc. Third, most studies sequenced peripheral blood mononuclear cells (PBMC) without accounting for the different cell types in the mixture. Given the differences in DNA methylation across different cell types [17, 18], studies based on unsorted PBMC samples are prone to confounding. Though different immune responses convolute in the multi-levelled abnormalities in SSc, T lymphocytes play a disproportionate role in SSc [19].

We undertook this pilot study to investigate genome-wide methylation patterns in CD4+ T cells using whole-genome bisulfite sequencing (WGBS) to profile the methylation status of CpG, CHG and CHH cytosines, and to assess the potential of this platform to identify changes in methylation. We successfully identified T cell-specific aberrant methylation patterns in SSc. We then comprehensively explored the regulatory impacts of these epigenetic alterations on biological functions and diseases. We also inferred potential single nucleotide polymorphisms (SNP)-CpG interactions that implicated underlying genetic control of methylation status. Finally, we provide an application enabling visualization of regions and summaries of our results across the genome.

## Methods

### Study subjects and ethical considerations

Nine SSc patients and nine control subjects gave informed consent and were recruited from an ongoing SSc research cohort based at McGill University, Montreal, Canada. Of the nine SSc patients, none were on immunosuppressive drugs at the time of sampling (three were previously on methotrexate and mycophenolate but those medications had been discontinued for > 1 year).

### Cell purification and whole-genome bisulfite sequencing (WGBS)

Forty milliliters of blood were obtained from each study subject and processed fresh within 4 h of being drawn. CD4+ T cells were positively selected [anti-CD4 microbeads (Miltenyi Biotec) and auto-MACS] and their purity assessed with flow cytometry. Only samples with a purity > 95% were used for genomic DNA extraction and sequencing. The samples were processed using the in-house DNA isolation and Illumina HiSeq 4000 PE 100 WGBS workflows at the McGill University and Genome Quebec Innovation Centre. Quality control of the genetic materials was performed using fluorescence assay quantification, agarose gel electrophoresis and NanoDrop nucleic acid quantification to ensure sufficient quantity, quality and purity (Additional file 18:).

### Data processing and filtering

The WGBS data were aligned to the human genome GRCh37 (hg19) using the NovoAlign™ pipeline (http://www.novocraft.com/). For each cytosine retained for further analysis, coverage by both strands in the paired-end sequencing library was required. To ensure accuracy in estimation of methylation level, valid cytosines with good read depth were extracted for CpG, CHG, and CHH motifs respectively. For each valid CpG dinucleotide, the estimated methylation level was obtained after merging methylated and unmethylated read counts for the forward and reverse cytosines. Read depth was required to be deeper than 3 at both C/G or C/H sites and the between-site difference in empirical methylation β values was required to be less than 0.2. For each valid CHG/CHH, minimum read depth required for further analysis was 6. Genome-wide SNPs were identified from the same dataset using the Bis-SNP pipeline [20].

### Identification of differentially methylated regions

We used *bumphunter* version 3.3 [21] to identify DMRs in five sets of comparisons:

(i) SSc cases (*N* = 9) versus female controls (*N* = 4);

(ii) SSc cases (N = 9) versus all controls (N = 9);

(iii) Diffuse SSc cases (*N* = 6) versus female controls (N = 4);

(iv) Limited SSc cases (*N* = 3) versus female controls (N = 4);

(v) Diffuse SSc cases (N = 6) versus limited SSc cases (N = 3).

In all sets of comparisons, we adjusted for the additive effect of age within *bumphunter*. Moreover, in (ii), we additionally adjusted for the effect of gender.

To maintain statistical power, we restricted the analysis to regions with consistently high coverage across samples and imposed a minimum coverage rate on a per-CpG dinucleotide or per-CHG/CHH site basis. We imposed a minimum sample-level coverage rate for each site analyzed; minimum sample sizes for the analyses presented in (i)-(v) were 6/9, 4/6, 3/4 and 3/3, respectively. Cytosines were then clustered into regions with a maximum 200 bp gap between two cytosines in the same region. The total number of tests done for each of (i)-(v) was 392,810, 383,235, and 380,756 for CpG, CHG and CHH, respectively.

Regions with an adjusted *p* value (q-value) < 0.05 and an average methylation level difference > 0.2 reported by *bumphunter* were considered to be DMRs. A Bonferroni corrected *p*-value threshold of significance for five genome-wide analyses each comprising more than 10^6^ comparisons would require a significance threshold below 2 × 10^−7^, which would be very unlikely to achieve here given the sample size. For example, for a simple t-test we would require a standardized difference of 9.2 to obtain 90% power at this significance level; that is, the mean difference in methylation between SSc and controls would have to be nine times larger than the standard deviation. Thus, our results should be considered as preliminary and therefore we have placed most emphasis on the results of analysis (i) as they included all the female patients and avoided the potential confounding effect of sex.

### Multiple testing

We used the false discovery rate estimates from *bumphunter* to select DMRs, and added a filter requiring that the difference in methylation be at least 0.2. We also performed a permutation test of the primary analysis comparison between SSc cases (*N* = 9) versus female controls (*N* = 4). We randomly relabelled samples as SSc cases or controls, and then repeated the genome-wide identification of DMRs using *bumphunter*. We repeated the permutation and genome-wide analysis 40 times, and then counted in how many permutations a previously identified DMR was still identified as a DMR with an identical or smaller *p* value. We also compared the number of identified DMRs between the original data and the permutations.

### Annotation of DMR and functional analysis

Genomic context of each DMR was annotated by *annotatr* [22] based on the most recent annotations of human genome downloaded from the UCSC genome browser (http://hgdownload.soe.ucsc.edu/goldenPath/hg38/database/. Accessed 5 March 2019). All genes overlapping with DMR were regarded as DMGs. We performed functional analysis using Ingenuity Pathway Analysis [23] to investigate potential biological impacts through epigenetic alterations in these DMGs. Adjusted *p* values and averaged methylation level difference of DMRs were used to indicate the degree of discrepancy. For single-DMR genes, the averaged difference of the corresponding DMR represents the gene-level difference. For genes associated with more than one DMR, we calculated the average of the averaged difference of each DMR to represent overall methylation level difference. Genes with both hypermethylated and hypomethylated DMRs may therefore have had the differences neutralized. Identification of DMR and functional analysis were conducted on CpG, CHG and CHH separately.

### Detection of SNP-CpG associations

We explored short-range SNP-CpG associations around a selected subset of CpG-based DMR identified in the comparison between SSc cases (*N* = 9) versus female controls (*N* = 4) that could be deemed consequential for SSc. Within a window of ±5 kb around each CpG-based DMR (inclusive), we extracted all SNPs as candidates for cis-interaction with the methylation pattern of the DMR. Simultaneously, within each CpG-based DMR, we first regressed out the effect of age and obtained residual methylation level on each CpG dinucleotide. We then used a multivariate method (PCEV [24]) to test associations between residual methylation levels and the binary disease status. We followed this by examining the PCEV-derived variable importance measures to identify the dinucleotide most strongly associated with disease status in the DMR. We extracted the residual methylation level on this specific CpG dinucleotide as a representative of the DMR and performed linear regression with all candidate SNPs of the corresponding DMR. In total, linear associations between 599 DMR and 36,838 candidate SNPs were tested. Unadjusted *p* values were reported with adjusted *R*^2^ for each of the 36,838 SNP-CpG pairs. To adjust for multiple testing on this analysis would require appropriate adjustments for linkage disequilibrium and were not undertaken here.

## Results

### Clinical characteristics

We recruited nine SSc patients and nine controls. The characteristics of the cases and controls are presented in Table 1. All cases and 4/9 controls were female. SSc disease duration was 10.4 ± 7.0 years, 6 SSc patients had diffuse and 3 had limited cutaneous skin involvement. None of the SSc patients were on immunosuppressive treatment at the time of sampling.

**Table 1:** Clinical characteristics

	SSc (*N* = 9)		Controls (*N* = 9)	
	Mean or %	SD or N	Mean or %	SD or N
Age, years	52.8	16.2	45.2	20.0
Female, %	100%	9	44.4%	4
Ethnicity, %				
Caucasian	77.8%	7	66.7%	6
Asian	22.2%	2	22.2%	2
Other	–	0	11.1%	1
Smoking, %				
Current	11.1%	1	–	0
Past	22.2%	2	22.2%	2
Never	55.6%	5	77.8%	7
Unknown	11.1%	1	–	0
Disease duration, years	10.4	7.0		
Interstitial lung disease, %	11.1%	1		
Arthritis, %	11.1%	1		
Myositis, %	22.2%	2		
Raynaud’s, %	100%	9		
Anti-nuclear antibodies				
Titer ≥1:80, %	100%	9		
Titer ≥1:160, %	66.7%	6		
Titer ≥1:640, %	55.6%	5		
Blood biochemical indices				
C-reactive protein (CRP), mg/L	29.5	65.3^¶^		
Erythrocyte sedimentation rate, mm/hr	23.7	14.1		
Abs. whole blood cell (WBC), K/ *μ* L	8.2	4.6		
Abs. lymphocytes, K/ *μ* L	1.6	0.7		
Abs. monocytes, K/ *μ* L	0.7	0.7^¶^		
Disease-specific variables				
Limited skin disease	33.3%	3		
Diffuse skin disease	66.7%	6		
Immunosuppressive medication^§^, %	33.3%	3		

^¶^ Over-dispersion due to extreme values

^§^ Methotrexate and/or Mycophenolate

### Widespread DNA methylation differences

Our WGBS captured methylation levels at 3,690,885 CpG dinucleotides with high coverage (Additional file 17: Figure S1), which largely exceeds studies using targeted sequencing technologies or BeadChip arrays. We were also able to extensively profile methylation patterns at 8,047,371 CHG and 17,331,920 CHH sites.

Our first (and primary) analyses compared methylation patterns between SSc patients and female controls while adjusting for the effect of age. We identified 599 regions genome-wide that exhibited differential CpG methylation under our criteria of a mean methylation β value difference greater than 0.2 and a *bumphunter* adjusted *p*-value < 0.05. These differentially methylated regions (DMRs) exhibited high specificity as none of them was identified as DMR in more than six out of 40 permutation tests, i.e. all DMRs had an empirical *p* value ≤0.15 (Additional file 17: Fig. S2). Nevertheless, given the small sample size of this pilot study, results must be interpreted cautiously. Additional file 17: Figure S3 shows that the number of DMRs identified on each chromosome varied across permutations, and that the number identified in the primary analysis tended to be higher than most of the permutations.

**Fig. 1:**
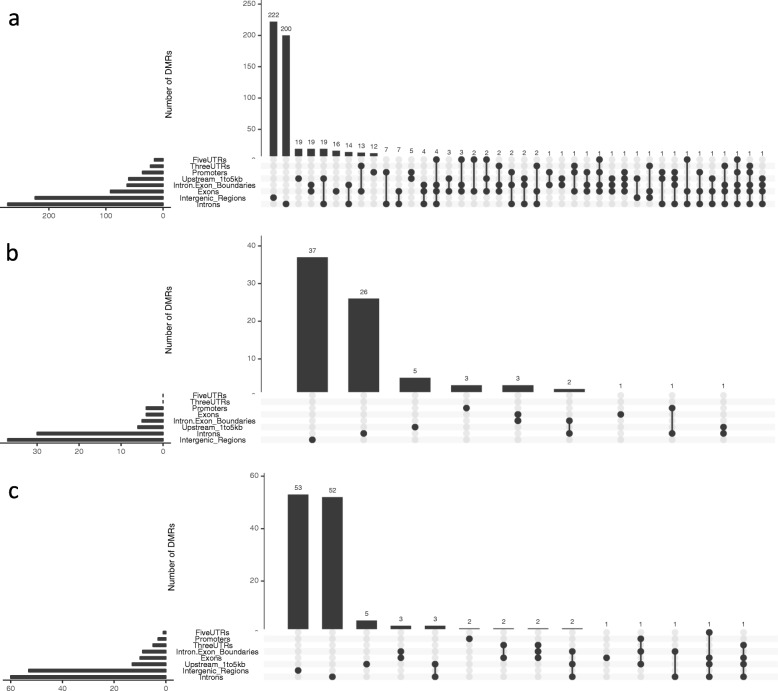
Genomic distribution of DMRs. DMRs based on (**a**) CpG, (**b**) CHG and (**c**) CHH were annotated to the up-to-date human genome separately. The heights of the bars in the graph indicate numbers of DMRs identified with a given genomic annotation configuration. The x-axis displays different annotation configurations. The dots under the bar graph together with the adjacent horizontal histogram display the configurations and their frequency. FiveUTR: 5′ UTR; ThreeUTR: 3′ UTR; Promoter: < 1 kb upstream of the transcription start site (TSS); Upstream_1to5kb: 1-5 kb upstream of the TSS; Intergenic_Region: > 5 kb upstream of the TSS. For example, in (**a**), the rightmost element in the display shows that 1 DMR was identified spanning at least one of each of these elements: 1-5 kb upstream region, exon, intron-exon boundary, and intron

These 599 DMR regions overlapped with 340 genes [differentially methylated genes (DMGs)], among which 169 showed hypermethylation and 163 showed hypomethylation in SSc (Additional file 1: Table S1). In addition, eight genes were identified as containing both hypermethylated and hypomethylated DMRs. Likewise, we also identified 79 CHG-based DMRs annotated to 39 genes (19 hypermethylated and 20 hypomethylated in SSc; Additional file 2: Table S2), as well as 129 CHH-based DMR annotated to 69 genes (28 hypermethylated and 41 hypomethylated in SSc; Additional file 3: Table S3). These three groups of CpG-, CHG- and CHH-based DMGs barely overlapped with each other (Additional file 17: Figure S4). We further inspected the genomic distribution of DMR and found that they were predominantly in intergenic and intronic regions, and relatively sparse in coding and regulatory regions (Fig. 1).

In contrast to most published work on WGBS, we were also able to examine the methylation on the X chromosome since this comparison involved only female participants. We identified 12 DMGs on the X chromosome, each of which contained one DMR (Table 2).

**Table 2:** DMG on X chromosome

Gene	Coordinates of DMR (GRCh37)	FDR	Averaged difference	Annotation
FTSJ1	[48,334,723, 48,334,739]	0.014	−0.23^¶^	5’UTR; Exon
MIR4770	[6,303,169, 6,303,169] ^§^	0.015	−0.37	Upstream 1-5 kb
PQBP1	[48,755,311, 48,755,329]	0.016	0.23	Promoter; 5’UTR; Exon; Intron
FIRRE	[130,880,912, 130,880,927]	0.021	−0.25	Intron
PCDH19	[99,663,316, 99,663,316] ^§^	0.027	−0.34	Exon
MECP2	[153,362,114, 153,362,135]	0.027	0.23	Intron
MIR363	[133,306,880, 133,306,910]	0.028	0.23	Upstream 1-5 kb
H2BFWT	[103,267,866, 103,267,866] ^§^	0.029	0.34	Exon
TIMM8A	[100,603,892, 100,603,909]	0.030	0.23	5’UTR; Exon; Intron-Exon Boundary
HTR2C	[113,818,760, 113,818,778]	0.035	0.21	5’UTR; Exon
TENM1	[123,994,369, 123,994,369] ^§^	0.044	−0.32	Intron
DCAF12L2	[125,300,434, 125,300,434] ^§^	0.046	−0.32	Promoter

^¶^ Negative value indicates hypomethylation in SSc

^§^ Single-dinucleotide DMRs were labelled by coordinates of the first C/G sites

In supplementary tables (Additional file 4: Tables S4, Additional file 5: Tables S5, Additional file 6: Tables S6), we also report the DMG identified using the analysis that included the male controls while adjusting for gender. Of note, including the male subjects gave slightly different DMG results from those presented using only females; however, the enriched pathways led to generally consistent interpretations (data not shown). We also investigated the difference between SSc subtypes. The supplement also provides the estimated DMG for diffuse SSc cases vs. controls, limited SSc cases vs. controls, and diffuse SSc cases vs. limited SSc cases (Additional file 7: Tables Tables S7, Additional file 8: Tables S8, Additional file 9: Tables S9, Additional file 10: Tables S10, Additional file 11: Tables S11, Additional file 12: Tables S12, Additional file 13: Tables S13, Additional file 14: Tables S14 and Additional file 15: Tables S15). Here, we observed that differential methylation might contribute to diffuse SSc and limited SSc in different ways since the DMGs identified in these three comparisons showed little overlap (Additional file 17: Fig. S5).

### Functional analysis

**Fig. 2:**
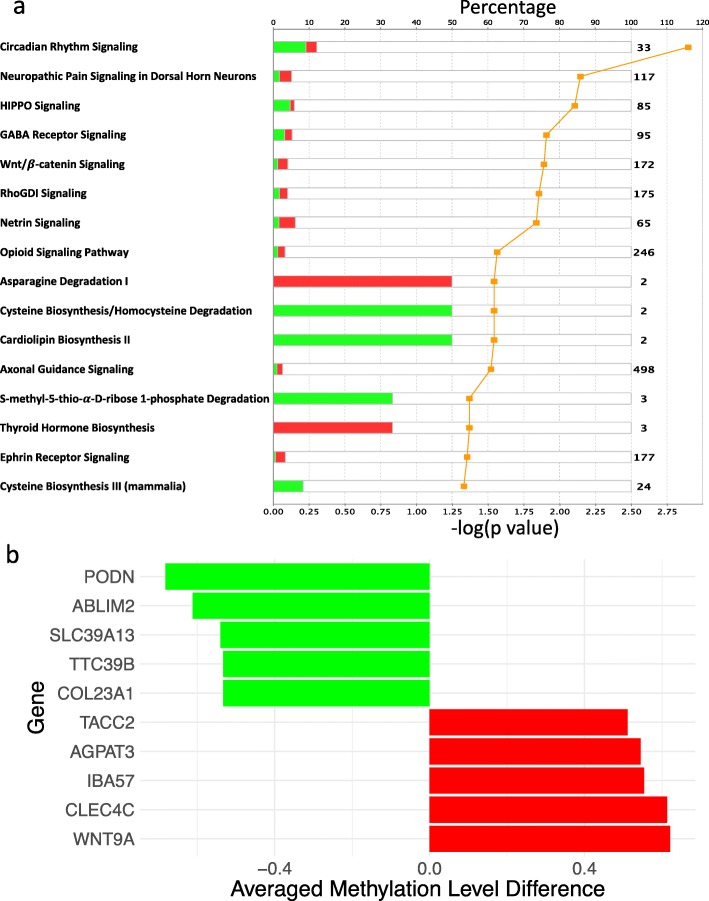
Biological impacts of differential methylation. (**a**) Significantly enriched canonical pathways (*p* < 0.05) based on CpG-DMRs. Pathways are sorted by *p* values. Percentage (indicated by bars) represents the proportion of significantly hypermethylated (red) and hypomethylated (green) genes among all genes in the corresponding pathway. Log-transformed p values are denoted by the orange line. (**b**) Top 10 differentially methylated genes with largest averaged difference in methylation levels in CpG-DMRs related to skin lesions. Five most hypermethylated genes (red) and five most hypomethylated genes (green) in SSc are illustrated

By functional enrichment analysis, we found that the 340 CpG-based DMGs were significantly associated with various signaling pathways, including HIPPO, Wnt/ *β*-catenin, RhoGDI, Netrin and Ephrin Receptor signaling (Fig. 2a). We also found CpG-based DMGs were associated with a wide variety of biological functions and multi-system diseases, including connective tissue disorders (Table 3). “Skin lesions” was significantly enriched in “diseases and biological functions” (*p* = 1.2 × 10^−13^). To illustrate the richness of this preliminary data, we highlighted the 10 most significantly differentially methylated genes associated with “skin lesions” in Fig. 2b.

**Table 3:** Top five networks influenced by CpG differential methylation

Network	Molecules in Network	Score	Focus Molecules
Embryonic Development, Organismal Development, Reproductive System Development and Function	AGO2, AP2A2, BOP1, CACHD1, CENPS/CENPS-CORT, CEP120, Ck2, CSNK1D, CSNK1E, ESR1, FAAP20, FAM83D, FAN1, FBXW11, FGFR1OP, HPCAL1, Hsp70, INPP5B, KTN1, MAP3K9, mir-363, NIN, phosphatase, PPFIBP1, PSD4, RAPGEF5, RBM19, RNF166, Rnr, SAMD11, Smad2/3, Ubiquitin, UMODL1, USP2, WRAP73	52	29
Cancer, Connective Tissue Disorders, Organismal Injury and Abnormalities	Akt, ANKRD11, CEACAM3, CHIA, COL23A1, COL4A1, COL5A1, collagen, Dynamin, Eph Receptor, EPHA1, EPHA10, GNB1L, GTPase, HOOK2, JINK1/2, KIAA1217, KIF26B, KSR1, Laminin (family), LIMS1, MAGI1, MTORC2, NAV1, NCK, NCK2, PARVB, PBXIP1, PI3K p85, PRDM16, PTPRN2, RPTOR, SH3BP4, SH3PXD2B, SH3RF3	42	25
Cell Morphology, Cellular Assembly and Organization, Cellular Development	BCAR3, CK1, CLEC4C, DNAJC2, ERK1/2, Fgf, FXN, GALNT2, GSPT1, Hdac, HSP, HSPA12B, KCNN2, KCNN3, KCNQ3, LIN54, MARCH1, MHC Class II (complex), MYL12A, Ngf, NTF3, PLC gamma, potassium channel, Ral, RALGDS, ROBO1, ROR2, SH3BP2, SLC7A8, TFDP1, TUSC3, Vla-4, Wnt, WNT9A, histone deacetylase	35	22
Gene Expression, Connective Tissue Disorders, Immunological Disease	CBS/CBSL, Ctbp, CTBP2, ETV6, GATAD2B, GPC6, Growth hormone, HDAC4, HDL-cholesterol, hemoglobin, HIPK2, HISTONE, Histone h4, Immunoglobulin, Jnk, KCNJ6, LDL, LDL-cholesterol, MECP2, MTHFD1L, N-cor, NCOR2, NFATC1, NPC2, Nr1h, NTM, PF4, Pias, PON1, POU2F1, SBNO2, STAT5a/b, TTC39B, VSX1, ZBTB16	33	21
Developmental Disorder, Hereditary Disorder, Organismal Injury and Abnormalities	Alpha tubulin, ANO1, ATP11A, ATP5MC2, BETA TUBULIN, caspase, Cyclin E, DLGAP1, DLGAP2, DYSF, ERK, FMN1, FTSJ1, Hsp27, Hsp90, Insulin, KCNG2, MAP3K20, Mek, NLRP12, NNAT, p70 S6k, PACRG, PARP, Pde, PDE9A, PIWIL1, Ppp2c, PRKN, Proinsulin, RGPD4 (includes others), Sos, TRPV2, XAF1, ZBTB17	31	20

The top five diseases and biological functions influenced by CHG differential methylation included projection of axons, similar to CpG-based DMGs, and formation of tight junctions (Additional file 17: Fig. S6). CHH-based DMGs were associated with cancer, as well the skeletal system and connective tissue disorders (Table 4).

**Table 4:** Top five diseases influenced by CHH differential methylation

Disease	Molecules related to Disease	FDR	Molecules
Familial skeletal dysplasia	ADAMTS2, DDR2, FAM20C, FDFT1, MYO18B, PDE4D, ROR2, TNFRSF11A	3.06E-07	8
Large intestine adenocarcinoma	ADAMTS2, AGT, ATXN3L, BAIAP2L1, CCDC155, CCDC85C, CFAP299, CMIP, CNNM2, CTDP1, DDR2, DNAJB13, FAM20C, FDFT1, FPR3, GAS7, GCM1, GRID2, IL13, IL27, IQCE, ITPK1, KCNJ12, KRT38, MCEE, MYO18B, NAV2, NDUFA10, NPHP4, PCSK6, PDE4D, PGS1, PITPNC1, PLEKHF1, PLEKHM3, PRKCA, PTPRN2, RALGPS2, RASA3, RIMBP2, RIN2, ROR2, RPS19, SLC30A1, SLC6A12, SPATS2L, SPG7, SV2C, TDRD5, TMEM92, TNFRSF11A, ZFYVE28	2.34E-06	52
Hereditary connective tissue disorder	ADAMTS2, AGT, CTDP1, DDR2, FAM20C, FDFT1, JDP2, MYO18B, PDE4D, RIN2, ROR2, TNFRSF11A	2.76E-06	12
Abdominal adenocarcinoma	ADAMTS2, AGT, ATXN3L, BAIAP2L1, CCDC155, CCDC85C, CFAP299, CMIP, CNNM2, CPNE6, CTDP1, DDR2, DNAJB13, FAM20C, FDFT1, FPR3, GAS7, GCM1, GRID2, IL13, IL27, IQCE, ITPK1, KCNJ12, KRT38, MCEE, MYO18B, NAV2, NDUFA10, NPHP4, PCSK6, PDE4D, PGS1, PITPNC1, PLEKHF1, PLEKHM3, PRKCA, PTPRN2, RALGPS2, RASA3, RIMBP2, RIN2, ROR2, RPS19, SLC30A1, SLC6A12, SPATS2L, SPG7, SSR1, SV2C, TDRD5, TMEM92, TNFRSF11A, ZFYVE28	8.89E-05	54
Liver carcinoma	ADAMTS2, AGT, CNNM2, CTDP1, DDR2, DNAJB13, FAM20C, FPR3, GAS7, GRID2, IL27, IQCE, JDP2, KCNJ12, MYO18B, NAV2, NDUFA10, NPHP4, PDE4D, PGS1, PITPNC1, PTPRN2, RALGPS2, RASA3, RIMBP2, RIN2, SLC30A1, SLC6A12, TDRD5, TNFRSF11A, ZFYVE28	2.32E-04	31

### Exploratory analysis of SNP-CpG associations

**Fig. 3:**
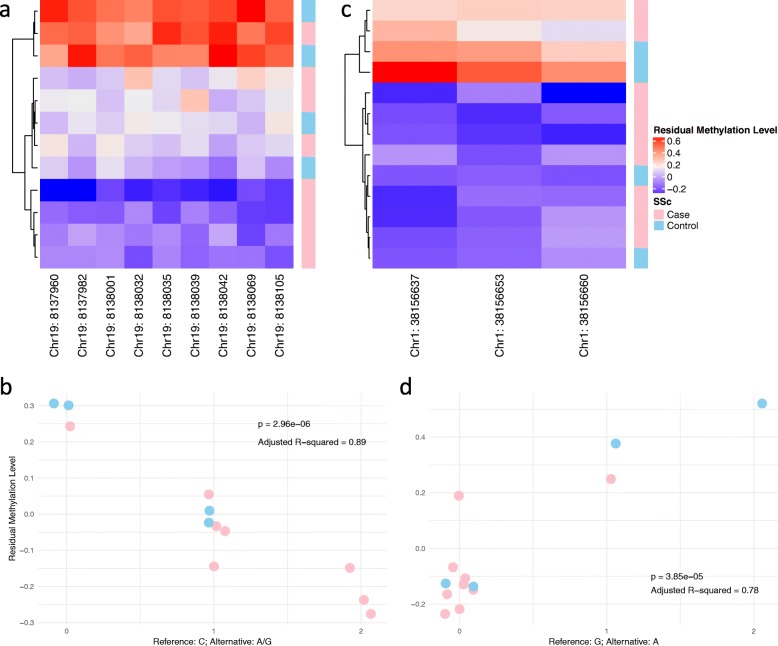
Illustration of SNP-CpG associations. (**a**) Methylation pattern of a nine-CpG DMR in FBN3. One SSc case was removed due to missing data. Methylation patterns exhibited a three-level stratification in this DMR where SSc cases were more prone to be hypomethylated. (**b**) Association between a C-to-A/G SNP at chr19:8138054 and significant loss of methylation in the DMR displayed in (**a**). (**c**) Methylation pattern of a three-CpG DMR in CDCA8. Methylation patterns exhibited a two-level stratification where SSc cases were more prone to be hypomethylated in this DMR. (**d**) Association between a G-to-A SNP at chr1:38156902 and significant increase in methylation in the DMR displayed in (**c**). Two SSc cases and one control with top three highest levels of methylation carried the A allele

We explored potential associations between SNPs and CpG-based DMR. Though we noticed a point of inflexion around a p value of 0.1 in the QQ-plot (Additional file 17: Fig. S7), we were aware that the small sample size and strong linkage disequilibrium could result in inflation of significant *p* values. Thus, we imposed a more stringent threshold requiring significant SNP-CpG pair to show a p value less than 5 ⨉ 10^−5^ and an adjusted *R*^2^ higher than 0.7. We identified 238 significant SNP-CpG associations, where the involved CpG-based DMRs were distributed across 54 genes (Additional file 16: Table S16). To illustrate typical associations arising from this analysis, Fig. 3 shows methylation, genotype and phenotype at two multi-CpG DMRs in genic regions, namely FBN3 and CDCA8. These analyses can generate hypotheses for how genetic, epigenetic and phenotype data may interact. However, our sample size was not sufficiently large to formally test 3-way interactions.

### Viewer of DMR results

Results at each DMR have been deposited online in Supplementary Tables, and we also provide a script (available at https://github.com/tianyuan-lu/SclerodermaMethylation/) to facilitate viewing of CpG methylation patterns.

## Discussion

In this pilot study, we identified DMRs and DMGs in each cytosine context (CpG, CHG and CHH) in SSc. We characterized the widespread functional impacts of differential methylation and potential genetic controls through SNP-CpG interactions.

Our findings regarding CpG-based differential methylation highlighted the role of signaling pathways implicated in the pathogenesis of SSc, including HIPPO [25], Wnt/ *β*-catenin [26–28], RhoGDI [29], Netrin [30] and Ephrin Receptor signaling [31]. Our findings were also consistent with the expected direction of effect. For example, hypomethylation of COL23A1, which codes for collagen XXIII and is expressed across different tissues including the skin and lungs [32], may be relevant to the excessive accumulation of collagen in this disease. COL23A1 has been previously reported to be hypomethylated in dermal fibroblasts of patients with SSc [33]. On the other hand, contrary to previous reports of over-expression of WNT9A in an animal model of SSc [34], we found that it was hypermethylated. Other genes illustrated in Fig. 2b could provide important novel targets of interest.

Abnormal expression of transforming growth factor beta (TGF-β) in SSc fibroblasts is central to disease pathogenesis [35, 36]. In our study of CD4+ T cells, we did not find significant changes in DNA methylation or in pathway analysis of TGF-β genes. However, we identified significant hypomethylation of SMAD3, which is a key signal transducer in the TGF-β signaling pathway and is responsible for maintaining CD4+ T cell homeostasis, particularly by inhibiting T cell receptor-induced naïve CD4+ T cell proliferation [37]. We posit this suggests linked yet different roles of TGF-beta dysregulation in SSc-CD4+ T cells and fibroblasts.

Several type I interferon (IFN) signaling pathway-associated genes were previously found to be hypomethylated in CD4+ and CD8+ T cells in SSc patients [38]. Although IFN-related pathways were not enriched in our pathway analyses, possibly due to the abundance of target genes in other pathways, our results still confirmed the importance of IFN signaling in the pathogenesis of SSc. For instance, we found that MX1 was hypomethylated in diffuse SSc patients (Additional file 7: Table S7) compared to healthy controls and that PARP11 was hypomethylated in limited SSc patients (Additional file 8: Table S8). Since hypomethylation of some of these IFN-related genes has been verified to have a strong impact on gene expression [38], it may be promising to further develop efficient biomarkers associated with this pathway.

In previous studies of SSc, the X chromosome has been mostly overlooked. Since our study consisted of mostly female subjects, we had an opportunity to investigate DMG on the X chromosome. Interestingly, we identified three significant DMG that were closely related to epigenetic modification, namely FTSJ1, coding for 2′-O-methyltransferase [39], FIRRE, a long non-coding RNA shown to be associated with histone H3 lysine 27 trimethylation [40], and MECP2, coding for methyl CpG binding protein 2 and regulating gene expression by modifying chromatin [41]. Interestingly, MECP2 has been reported to be involved in SSc skin fibrosis [42]. Aberrant methylation patterns in these epigenetic regulators suggests that the epigenetic regulatory mechanism in SSc is more complex and hierarchical than previously appreciated. Our findings regarding DNA methylation on the X chromosome were different from those of an earlier study [43]. However, in that study, methylation levels were assessed using peripheral blood mononuclear cells. We posit that our study reduced the confounding effect arising from cell mixtures.

Apart from CpG-based differential methylation, our study identified novel insights into the contribution of CHG and CHH methylation in SSc. For example, CCR3 has been previously reported to be increased in SSc monocytes [44]. We identified other genes with CHH-based DMR that could contribute to fibrosis and angiogenesis, including ADAMTS2 [45] and DDR2 [46–48]. All these findings suggest the role of non-CpG methylation that is worthy of further study.

Genome-epigenome interaction is a crucial component of regulation of gene expression and its importance is best established in the pathogenesis of cancer [49, 50]. A previous study reported that the SNP rs17435 linked to MECP2 (mentioned above) was related to the clinical outcome of SSc [51]. This suggests that genome-epigenome interaction could be a key to understand aberrant gene regulation in SSc. Our study pinpointed 238 potential short-range SNP-CpG pairs where the methylation levels were strongly associated with the genotype. As illustrated in Fig. 3, differential methylation in FBN3, a gene that codes for extracellular matrix macromolecules responsible for architectural functions in connective tissues [52, 53], was associated with a SNP at chr19:8138054 (rs7257948). Similarly, differential methylation in the cell division cycle associated 8 (CDCA8) gene, which plays an important role in mitosis [54] and has been implicated in SSc [55], was associated with a SNP at chr1:38156902 (rs3762352). We suggest that differential methylation in these genes may be controlled by genetic mutations and may serve as a mediator towards modulation of gene expression [56–58]. It is beyond the scope of this study to investigate these functional effects, although these could be the subject of future research.

We acknowledge that the small sample size and possible confounding due to differences in the characteristics of the cases and controls (Table 1) are important limitations of our study. For instance, in this study, we did not identify significant differential methylation in the HLA genes, where known genetic variants are associated with the pathogenesis of SSc [59]. Regardless, this result could not refute that differential epigenetic modifications might be dependent on specific haplotypes, since the small sample size is not able to support statistical tests for differences at such a calibrated level. However, this study was planned and executed as a pilot to explore the potential of WGBS for assessing genome-wide methylation in SSc. Since adjusting for confounders could have led to overfitting of our statistical models, we focused our primary analyses on the SSc cases (who were all female) and the female controls. Additional analyses including male controls, while adjusting for the fixed effect of sex (Additional file 4: Tables S4, Additional file 5: Table S5 and Additional file 6: Table S6) and comparisons between different SSc subtypes (Additional file 7: Tables S7, Additional file 8: Tables S8, Additional file 9: Tables S9, Additional file 10: Tables S10, Additional file 11: Tables S11, Additional file 12: Tables S12, Additional file 13: Tables S13, Additional file 14: Tables S14 and Additional file 15: Tables S15) are provided in the supplement. We have deliberately refrained from making further interpretations of these additional comparisons to minimize overinterpretation of this analysis. Nonetheless, our study forms a foundation for future studies with larger cohorts. Our results and the scripts we have provided for browsing them can be used to confirm previous or future findings, or to explore how other risk factors interact with epigenetic modifications to promote pathogenesis in different subtypes of SSc.

## Conclusion

By profiling genome-wide DNA cytosine methylation landscape in SSc CD4+ T lymphocytes, we found widespread differential methylation involving genes relevant to disease pathogenesis. Some of the abnormal DNA methylation patterns in SSc patients are also associated with neighboring genetic variation. These findings can provide a good source for identifying novel targets of interest in SSc, developing well-profiled epigenetic biomarkers that may supplement current diagnostic and prognostic tests, as well as profoundly investigating genome-epigenome interaction in SSc pathogenesis and progression.

## Supplementary information

**Additional file 1: Table S1.** Summary of CpG-based DMGs.

**Additional file 2: Table S2.** Summary of CHG-based DMGs.

**Additional file 3: Table S3.** Summary of CHH-based DMGs.

**Additional file 4: Table S4.** Summary of CpG-based DMGs adjusted for sex effect.

**Additional file 5: Table S5.** Summary of CHG-based DMGs adjusted for sex effect.

**Additional file 6: Table S6.** Summary of CHH-based DMGs adjusted for sex effect.

**Additional file 7: Table S7.** Summary of CpG-based DMGs between diffuse SSc and controls.

**Additional file 8: Table S8.** Summary of CpG-based DMGs between limited SSc and controls.

**Additional file 9: Table S9.** Summary of CpG-based DMGs between diffuse SSc and limited SSc.

**Additional file 10: Table S10.** Summary of CHG-based DMGs between diffuse SSc and controls. (XLS 32 kb)

**Additional file 11: Table S11.** Summary of CHG-based DMGs between limited SSc and controls.

**Additional file 12: Table S12.** Summary of CHG-based DMGs between diffuse SSc and limited SSc.

**Additional file 13: Table S13.** Summary of CHH-based DMGs between diffuse SSc and controls.

**Additional file 14: Table S14.** Summary of CHH-based DMGs between limited SSc and controls.

**Additional file 15: Table S15.** Summary of CHH-based DMGs between diffuse SSc and limited SSc.

**Additional file 16: Table S16.** Summary of SNP-CpG associations.

**Additional file 17: Figure S1.** Number of cytosines used in our analysis, i.e. passing quality filters for read depth and number of samples covered. **Figure S2.** Empirically estimated *p* values for CpG-based DMRs with a q value < 0.05 and with methylation differences larger than 0.2. **Figure S3.** Number of (a) largely differentially methylated regions (bumps) and (b) significant DMRs identified by bumphunter in original test and 40 permutation tests by chromosome. **Figure S4.** Overlap of CpG-, CHG- and CHH-based DMGs. **Figure S5.** Overlap of SSc clinical-type-specific DMGs. **Figure S6.** Top five enriched diseases and biological functions based on CHG-DMRs. **Figure S7.** Quantile-quantile plot of unadjusted p values obtained in 36,838 association tests for SNP-CpG associations.

**Additional file 18:** Supplementary Information. Quality control report of de-identified samples.

## Data Availability

The datasets generated and analysed during the current study are available in GitHub repository http://github.com/tianyuan-lu/SclerodermaMethylation.

## Abbreviations

DMG: Differentially methylated gene
DMR: Differentially methylated region
HLA: Human leukocyte antigen
PBMC: peripheral blood mononuclear cell
SNP: Single nucleotide polymorphism
SSc: Systemic sclerosis
TGF-β: Transforming growth factor beta
WGBS: Whole-genome bisulfite sequencing
