# Supplementary material for: Whole-genome bisulfite sequencing in systemic sclerosis provides novel targets to understand disease pathogenesis
Source: BMC Med Genomics. 2019 Oct 24;12:144. doi: 10.1186/s12920-019-0602-8 (PMC6813992; doi:10.1186/s12920-019-0602-8)
Supplement: Supplementary file 17 — Additional file 17: Figure S1. Number of cytosines used in our analysis, i.e. passing quality filters for read depth and number of samples covered. Figure S2. Empirically estimated p values for CpG-based DMRs with a q value < 0.05 and with methylation differences larger than 0.2. Figure S3. Number of (a) largely differentially methylated regions (bumps) and (b) significant DMRs identified by bumphunter in original test and 40 permutation tests by chromosome. Figure S4. Overlap of CpG-, CHG- and CHH-based DMGs. Figure S5. Overlap of SSc clinical-type-specific DMGs. Figure S6. Top five enriched diseases and biological functions based on CHG-DMRs. Figure S7. Quantile-quantile plot of unadjusted p values obtained in 36,838 association tests for SNP-CpG associations. [file 12920_2019_602_MOESM17_ESM.pdf]

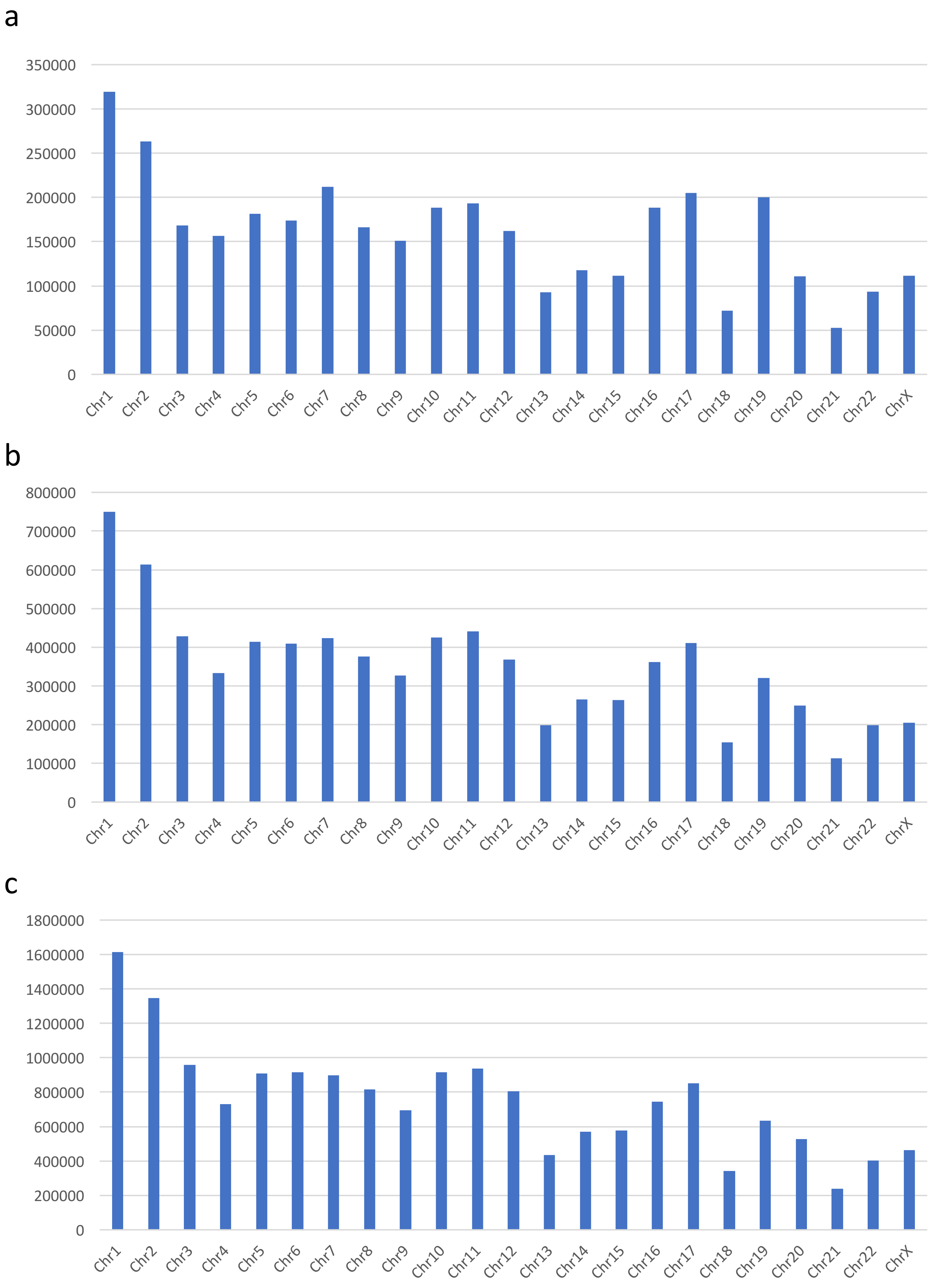

Figure S1. Number of cytosines used in our analysis, i.e. passing quality filters for read depth and number of samples covered. (a) 3,690,885 CpG dinucleotides, (b) 8,047,371 CHG sites and (c) 17,331,920 CHH sites. Coverage per chromosome was consistent for all three types of cytosine depending on chromosome size and GC content.

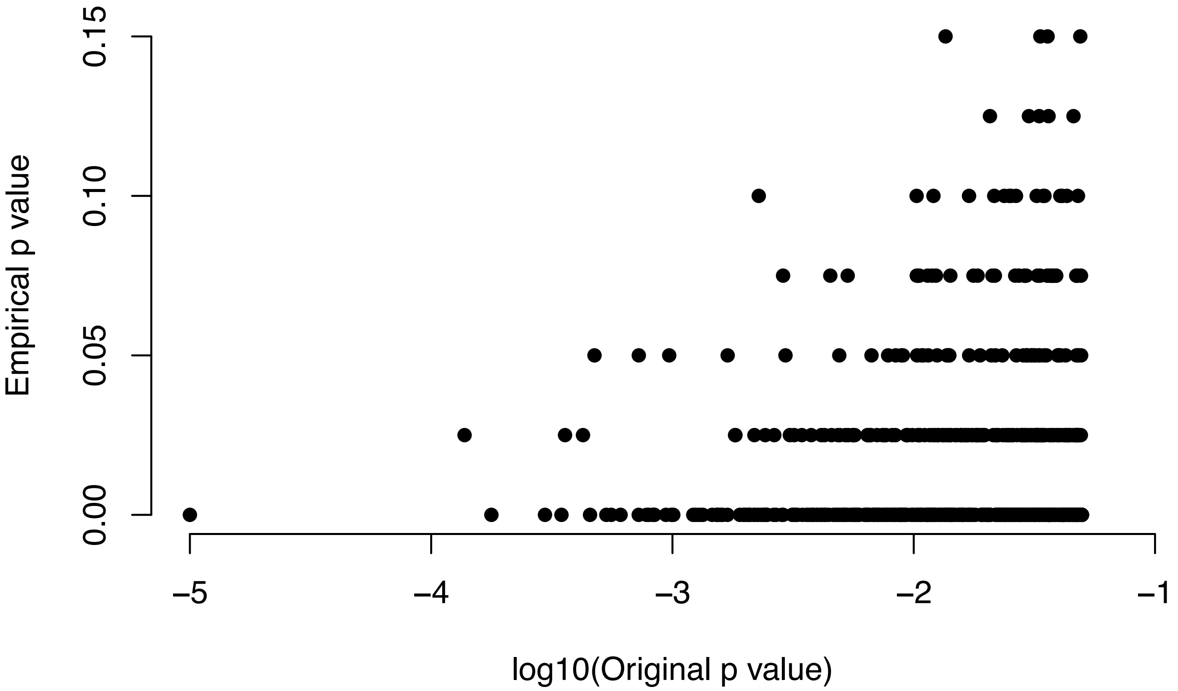

Figure S2. Empirically estimated p-values for CpG-based DMRs with q-value<0.05 and with methylation differences larger than 0.2. Estimates are obtained from 40 permutations of disease labels for each of the 599 DMRs identified, and this proportion is plotted against the log-transformed original p value. DMRs with smaller p values are more likely to have a smaller empirical p value.

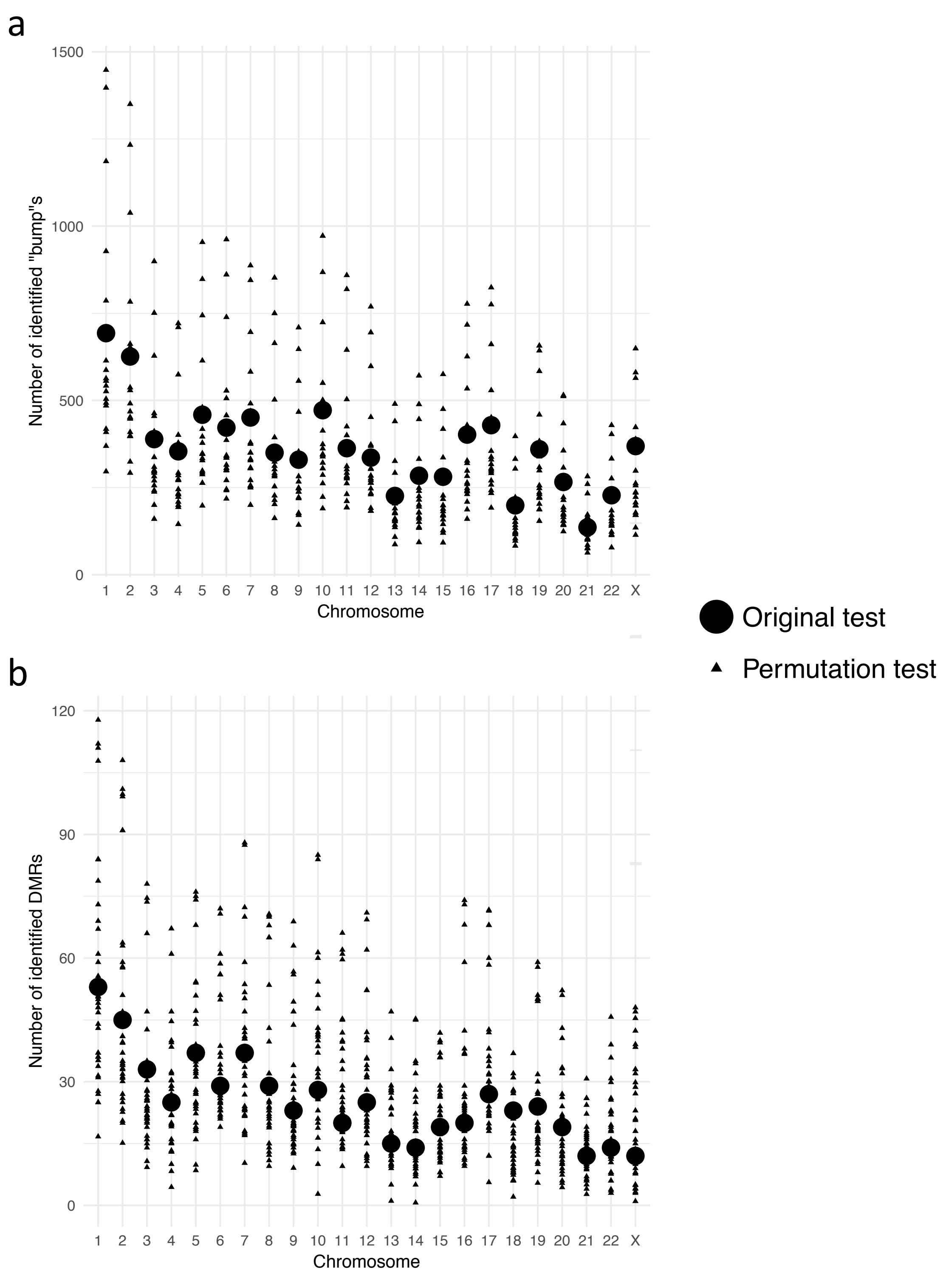

Figure S3. Number of (a) largely differentially methylated regions (bumps) and (b) significant DMRs identified by *bumphunter* in original test and 40 permutation tests by chromosome. Bumps had difference in methylation β value >0.2 between cases and controls in the original test or between pseudo-cases and pseudo-controls in permutation tests. Adjusted p values were reported only for bumps. DMRs were defined as bumps with an adjusted p value <0.05.

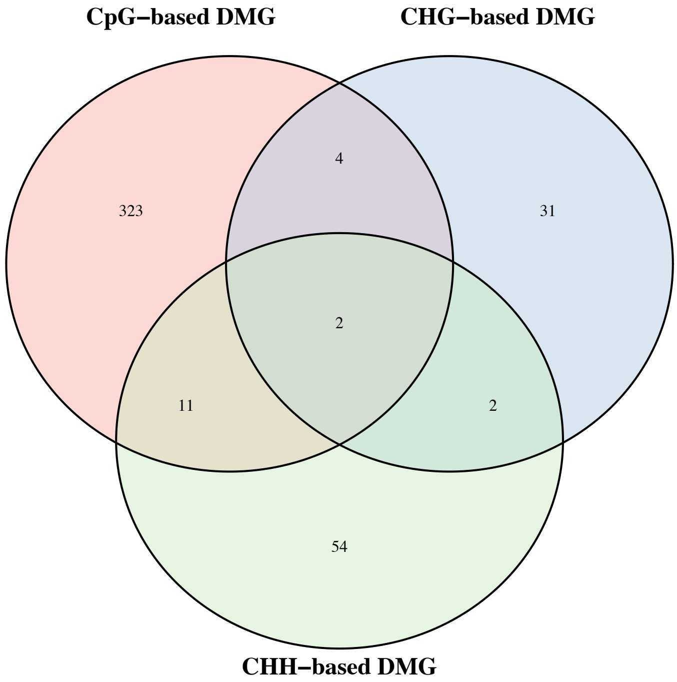

Figure S4. Overlapping of CpG-, CHG- and CHH-based DMGs. All genes with at least one DMR were considered DMGs. Most genes were only differentially methylated in one of the three cytosine contexts.

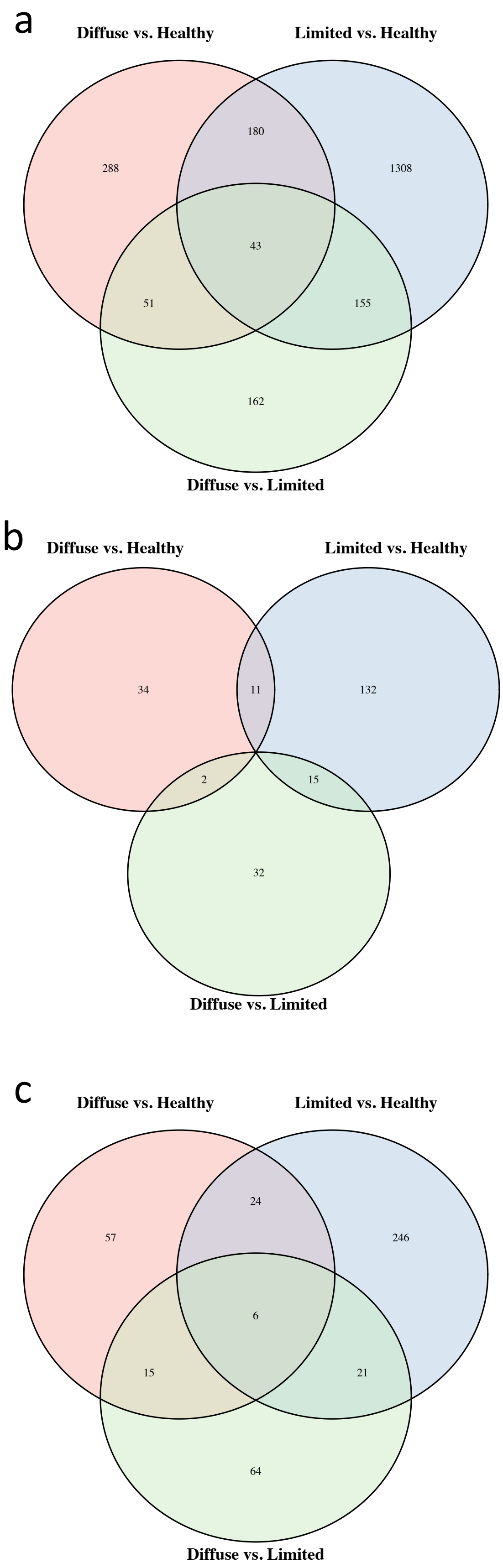

Figure S5. Overlap of SSc clinical-type-specific DMGs. DMGs based on (a) CpG, (b) CHG and (c) CHH were identified by pairwise comparisons among diffuse SSc cases, limited SSc cases and controls.

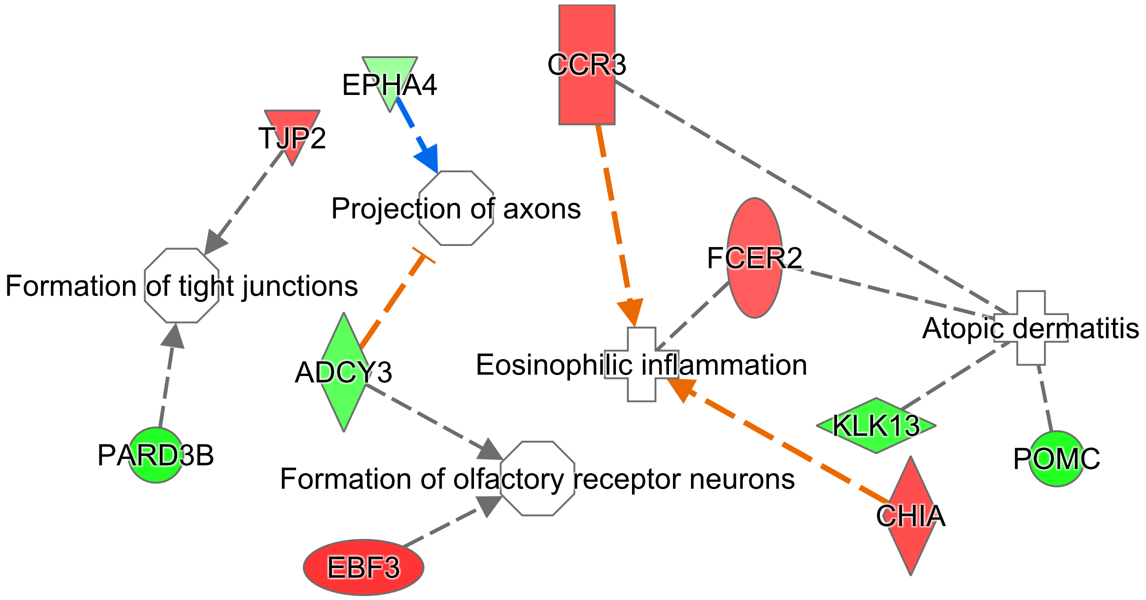

Figure S6. Top five enriched diseases and biological functions based on CHG-DMRs. Hypermethylated genes (red) and hypomethylated genes (green) in SSc are represented by nodes together with disease/function-related terms (white). Orange and blue arrays indicate existence of evidence in support of direct activation and inhibition, respectively.

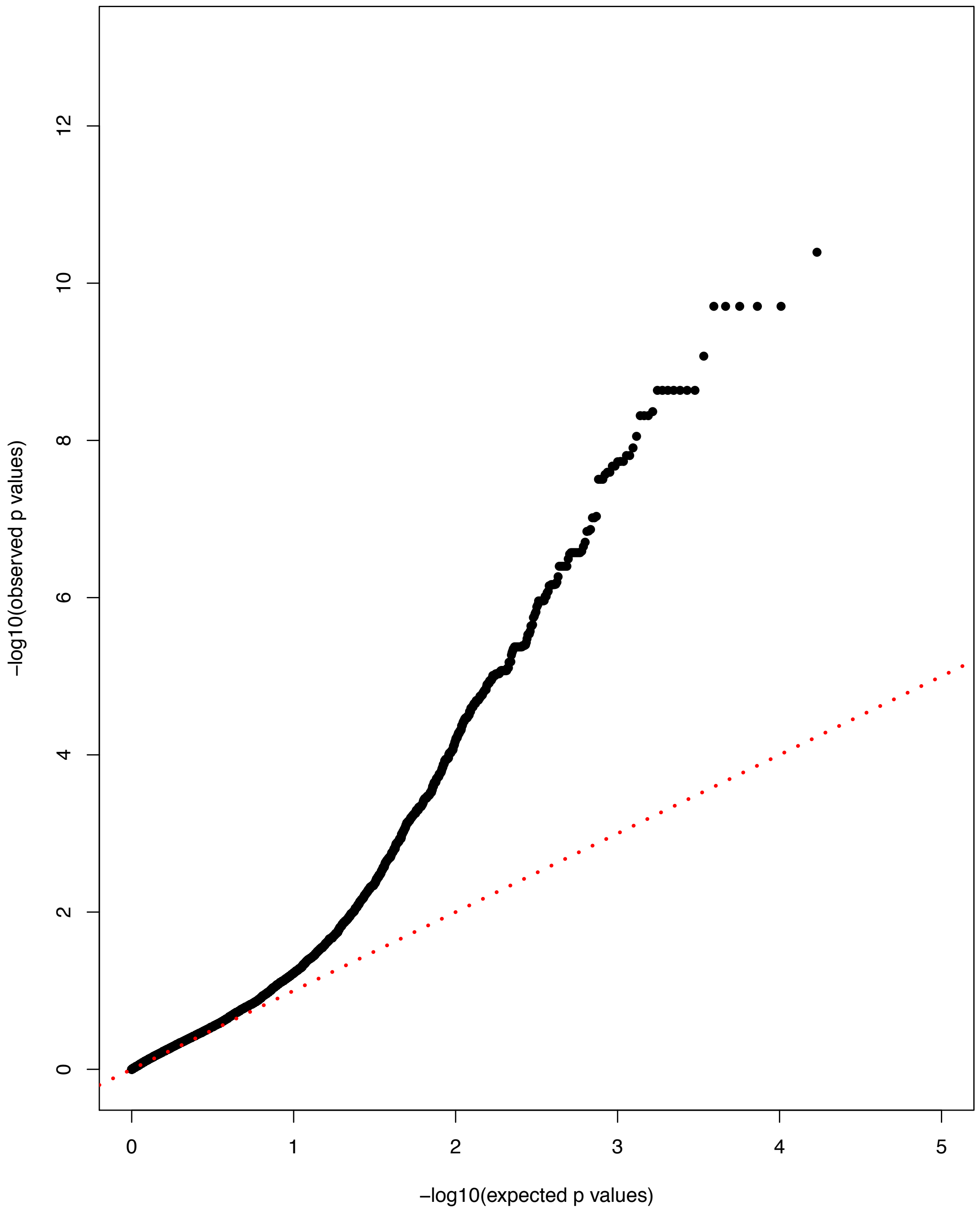

Figure S7. Quantile-quantile plot of unadjusted p values obtained in 36,838 association tests for SNP-CpG associations.
